# Interleukin-33 promoting Th1 lymphocyte differentiation dependents on IL-12

**DOI:** 10.1016/j.imbio.2015.11.013

**Published:** 2016-03

**Authors:** Mousa Komai-Koma, Eryi Wang, Mariola Kurowska-Stolarska, Dong Li, Charles McSharry, Damo Xu

**Affiliations:** a Institute of Infection, Immunity and Inflammation, University of Glasgow, Glasgow, UK; b Department of Haematology & Immunology, Faculty of Medicine, Umm Al-Qura University, Mecca, KSA, Saudi Arabia; c Department of Pharmacology, Medical Research Council Centre for Drug Safety Science, University of Liverpool, Liverpool, UK

**Keywords:** IL, interleukin, APC, antigen-presenting cells, RT, reverse transcription, DLNs, draining lymph nodes, IL-33, Th1 cell development, IFN-γ production

## Abstract

The pro-Th2 cytokine IL-33 is now emerging as an important Th1 cytokine-IFN-γ inducer in murine CD4^+^ T cells that is essential for protective cell-mediated immunity against viral infection in mice. However, whether IL-33 can promote human Th1 cell differentiation and how IL-33 polarizes Th1 cells is less understood. We assessed the ability of IL-33 to induce Th1 cell differentiation and IFN-γ production *in vitro* and *in vivo*. We report here that IL-33 alone had no ability in Th1 cell polarization. However it potentiated IL-12-mediated Th1 cell differentiation and IFN-γ production in TCR-stimulated murine and human CD4^+^ T cells *in vitro* and *in vivo*. IL-33 promoted Th1 cell development *via* MyD88 and synergized with IL-12 to enhance St2 and IL-12R expression in CD4^+^ T cells.

These data therefore provide a novel mechanism for Th1 cell differentiation and optimal induction of a Type 1 response. Thus, IL-33 is capable of inducing IL-12-dependent Th1 cell differentiation in human and mouse CD4^+^ T cells.

## 1 Introduction

The recognition that CD4^+^ T cells can be differentiated into functionally distinct subsets, including Th1 and Th2 cells, based on their distinct profile of cytokines to which they respond and secrete after stimulation, represented a major advance in immunology (Mosmann and Coffman, 1989, Sher and Coffman, 1992). Thus, IL-12 produced by antigen-presenting cells (APCs), together with TCR activation, drives the differentiation of CD4^+^ precursor T cells to Th1 cells which produce mainly IFN-γ and are important for defense against intracellular pathogens but also mediate auto-immune inflammation. IL-4 induces the differentiation of Th2 cells which produce mainly IL-4 and are associated with eradication of extracellular parasites but also mediate allergic inflammation (Mosmann and Coffman, 1989, Constant and Bottomly, 1997). Furthermore, Th1 and Th2 cells counter-regulate each other’s function by the relative concentration of the cytokines they produce. The balance of the Th1 and Th2 response frequently determines the outcome of several important infectious and autoimmune diseases (Seder et al., 1993, Constant and Bottomly, 1997, O’Garra et al., 1997). It is therefore of considerable importance to define the mechanisms underlying the preferential induction of each of these T cell subsets. The members of the IL-1 family including IL-1β and IL-18 are closely associated with Th1 and Th2 cell differentiation (Xu et al., 1998a, Xu et al., 1998b, Xu et al., 2000, Guo et al., 2009); we therefore investigated the role of IL-33 (Schmitz et al., 2005), a member of the IL-1 cytokine family, in the induction and regulation of Th1 cell development *in vitro* and *in vivo*.

IL-33 is only produced by innate immune cells including epithelial cells, DCs and macrophages (Schmitz et al., 2005, Rank et al., 2009, Pichery et al., 2012), and is released most probably when cells sense inflammatory signals or undergo necrosis (Moussion et al., 2008, Cayrol and Girard, 2009). IL-33 signals *via* its receptor ST2 and co-receptor IL-1R accessory protein (IL-1RAcP) (Schmitz et al., 2005, Ali et al., 2007, Chackerian et al., 2007). ST2 is expressed on a wide-range of innate immune cells (Schmitz et al., 2005, Trajkovic et al., 2004), but on limited adaptive immune cells primarily Th2 cells (Xu et al., 1998a, Xu et al., 1998b, Löhning et al., 1998). IL-33 is a potent inducer of type 2 cytokines including IL-5 and IL-13 by directly activating ST2 on innate immune cells and Th2 cells, and plays an important role in parasite infection, allergy and asthma (Schmitz et al., 2005, Komai-Koma et al., 2012, Saglani et al., 2013). However, recent study suggests that IL-33 is also able to promote Th1 development and function in mice (Baumann et al., 2015). We found previously that IL-33 can induce IFN-γ production in Th1-mediated inflammatory arthritis and hyper-nociception in mice (Xu et al., 2008, Verri et al., 2008). However, whether IL-33 can polarize human Th1 cells and the underlying mechanism by which IL-33 drives the Th1 development is less understood. We therefore studied the effect and mechanism of IL-33 on Th1 cell development in human and murine CD4^+^ T cells *in vitro* and *in vivo*.

## 2 Materials and methods

### 2.1 Mice

C57BL/6 mice were obtained from Harlan Olac (Bicester, Oxon, UK). St2*^−/−^* mice have been previously described (Kurowska-Stolarska et al., 2008). All mice were housed in specific-pathogen-free conditions at Glasgow University, UK, and mice of 5–6 weeks old were used in the experiments. Procedures were in accordance with the UK Home Office animal experimentation guidelines.

### 2.2 Recombinant IL-33

Recombinant IL-33 (rIL-33) was obtained from PeproTech and also expressed in *Escherichia coli* and purified by Ni-NTA affinity chromatography as described previously (Kurowska-Stolarska et al., 2008, Humphreys et al., 2008, Komai-Koma et al., 2012). Endotoxin was removed by purification with polymyxin B chromatography. The purity of rIL-33 was >97% by silver staining and endotoxin levels were <0.1 unit/μg of protein by the Limulus Amebocyte Lysate QCL-1000 pyrogen test (Cambrex). rIL-33 from PeproTech showed similar results.

### 2.3 Immunization and cytokine injection

Mice were immunized subcutaneously with 100 μl of chicken ovalbumin (OVA, Fraction V, Sigma–Aldrich) (130 μg) adsorbed to 1% alum (Brenntag Biosector) ±cytokines (1 μg/mouse), rIL-12 (PeproTech) or rIL-33 co-adsorbed to alum/OVA before inoculation into groups of mice. Boosting inoculations were performed in the same fashion 1 week later.

### 2.4 Cytokine measurement

Mouse draining lymph nodes (DLNs), spleen and blood were collected from mice at end of the experiments. Single cell suspensions from spleen or DLNs were cultured in 24-well plates at 4 × 10^6^ cells in 2 ml per well and stimulated with medium alone or with different dose of OVA peptide. After 72 h, supernatants were collected and concentrations of IFN-γ and IL-4 were measured by ELISA using paired antibodies according to the manufacturer’s instructions (R & D systems).

### 2.5 CD4^+^ T cell purification and culture

Human cord blood was obtained from informed consented mothers and peripheral blood mononuclear cells (PBMCs) were isolated by density gradient centrifugation through Lymphoprep (Nycomed). CD4^+^ T cells from human PBMC and murine spleen were purified by negative selection (AutoMACS; MiltenyiBiotec). T cells were cultured in RPMI 1640 supplemented with 10% FCS, 2 mM l-glutamine, 100 U/ml penicillin and 100 μg/ml streptomycin. Purified CD4^+^ T cells (purity ≥98%, 2 × 10^6^ cells/ml) were activated with plate-bound anti-CD3 Abs (3 μg/ml; BD Biosciences), rIL-12, and different doses of rIL-33 or a combination of these cytokines for different times as indicated. Freshly isolated cells, DLNs or spleen of immunized mice were cultured with different dose of OVA peptide without cytokines for 1–3 days. The cells, cellular RNA and culture supernatants were collected for analysis by flow cytometry, PCR or ELISA, respectively.

### 2.6 Flow cytometry

Cultured or freshly isolated cells from spleen were stimulated with PMA (500 ng/ml) and ionomycin (50 ng/ml; both from Sigma–Aldrich) for 4 h; GolgiStop was added during the final 3 h. The cells were incubated with anti-mouse CD16/32 to block non-specific Fc binding (BD Biosciences) followed separately by PerCP-conjugated anti-CD3, anti-CD4, (BD Biosciences) or appropriate isotype controls. Cells were then fixed with Cytofix/Cytoperm buffer (BD Biosciences), permeabilized with perm/wash buffer (BD Biosciences), and incubated with FITC-conjugated anti-IFN-γ, PE-conjugated anti-IL-4 (all from BD Biosciences) or isotype controls followed by incubation with secondary antibodies or streptavidin if necessary. The cells were analysed on dual laser (488 nm & 633 nm) FACSCalibur flow cytometer (Becton Dickinson, Mountain View, CA) using CellQuest Pro software (Becton Dickinson, Mountain View, CA).

### 2.7 Antibody measurement

Blood samples were taken from groups of mice 10 days after immunization with OVA with or without rIL-12 and rIL-33 adsorbed to alum. OVA-specific IgG1 and IgG2a levels in plasma were measured using OptEIA ELISA kits (BD Biosciences).

### 2.8 qPCR

RNA was purified from cultured cells or tissue samples using the RNeasy Mini Kit following the manufacturer’s instructions (Qiagen). Reverse Transcription (RT) of RNA into cDNA was carried out using High-Capacity cDNA Reverse Transcription Kits (Applied Biosystems). Real-time PCR was performed using Fast SYBR Green master mix on a Prism 7900HT (Applied Biosystems).

### 2.9 Statistical analysis

Statistical evaluation of cell proportions, cytokine production, and qPCR analysis was performed using the two-tail unpaired Student’s *T*-test. All data are expressed as mean ± SD. 5–7 mice/group/experiment. *p *< 0.05 was considered statistically significant. All experiments were repeated at least 3 times.

## 3 Results

### 3.1 IL-33 potentiates IL-12-mediated Th1 cell polarization *in vitro*

**Fig. 1:**
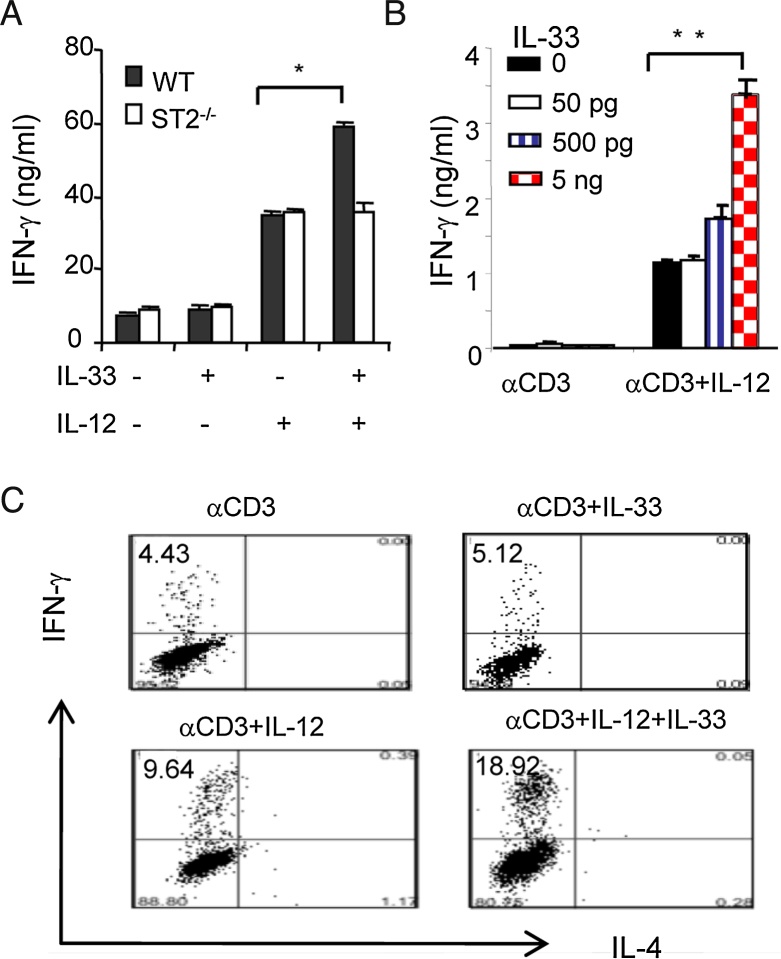
IL-33 enhances Th1 cell polarization *via* ST2 and IL-12. CD4^+^ T cells from WT, ST2^−/−^ mice (A) or human cord blood (B) were stimulated with anti-CD3 Abs with or without IL-33 (10 ng/ml) or IL-12(10 ng/ml) for 72 h. Supernatants IFN-γ and IL-4 concentrations were measured by ELISA. (C) Human CD4^+^ T cells were stimulated as above and intracellular cytokines were determined using FACScan. Data are presented as mean ± SD, *n* = 5 mice/group, and are representative of three independent experiments. ^∗^*p* < 0.05 compared with PBS control and ^**^p < 0.01 compared to IL-33 controls.

It was reported previously that IL-33 can polarize virus-specific Th1 cells in mice (Baumann et al., 2015). We found that IL-33 alone failed to induce IFN-γ secretion in CD4^+^ T cells *in vitro* (Fig. 1A). We sought next to investigate whether IL-33 and IL-12 can synergise in Th1 cell differentiation from naïve CD4^+^ T cells. To test this hypothesis, naïve CD4^+^ T cells isolated from WT or ST2*^−/−^* mice were cultured under Th1 cell polarization conditions with anti-CD3 antibody, with or without IL-12 or IL-33, alone or together, for 72 h. As expected, IL-12 stimulated higher levels of IFN-γ (Fig. 1A) but not IL-4 production as expected (data not shown) in polarized CD4^+^ T cells from WT and ST2*^−/−^* mice compared to cells cultured without IL-12 (Fig. 1A). IL-33 alone failed to stimulate the synthesis of both IFN-γ and IL-4 in WT and ST2*^−/−^* cells (Fig. 1A and data not shown). However, IL-33 further enhanced the synthesis of IFN-γ induced by IL-12 in an ST2-dependent manner (Fig. 1A). No IL-5, IL-13, IL-17 and IL-10 had been detected in the culture of IL-33 and IL-12 stimulated cells.

To determine if IL-33 and IL-12 are also capable of inducing the same cytokine response pattern in human T cells, antigen naïve human CD4^+^ T cells from cord blood were activated with anti-CD3 antibody in the presence or absence of human IL-12, and graded doses of IL-33 (0–5 ng/ml). Human T cells also produced enhanced levels of IFN-γ (Fig. 1B) but not IL-4 (data not shown) in responding synergistically to the combined stimulation of IL-12 and IL-33 compared to either IL-12 or IL-33 alone (Fig. 1B).

We further determined whether the IL-33 and IL-12 synergistically enhanced IFN-γ production in CD4^+^ T cells was due to enhancing Th1 cell polarization by examining their production of IL-4 and IFN-γ using intracellular cytokine staining and FACS analysis. Consistent with the cytokine ELISA data in Fig. 1B, IL-33 and IL-12 synergistically enhanced the population of human CD4^+^ T cell which selectively expressed IFN-γ but not IL-4 compared to the PBS control (Fig. 1C). Similar results were also obtained from murine CD4^+^ T cells (data not shown). These results suggest that IL-33 enhanced the polarization of Th1 but not Th2 cells in the presence of IL-12.

### 3.2 The mechanism by which IL-33 potentiates IL-12-mediated IFN-γ production

We next investigated the mechanism by which IFN-γ production is increased in the CD4^+^ T cells stimulated synergistically with IL-33 and IL-12.

**Fig. 2:**
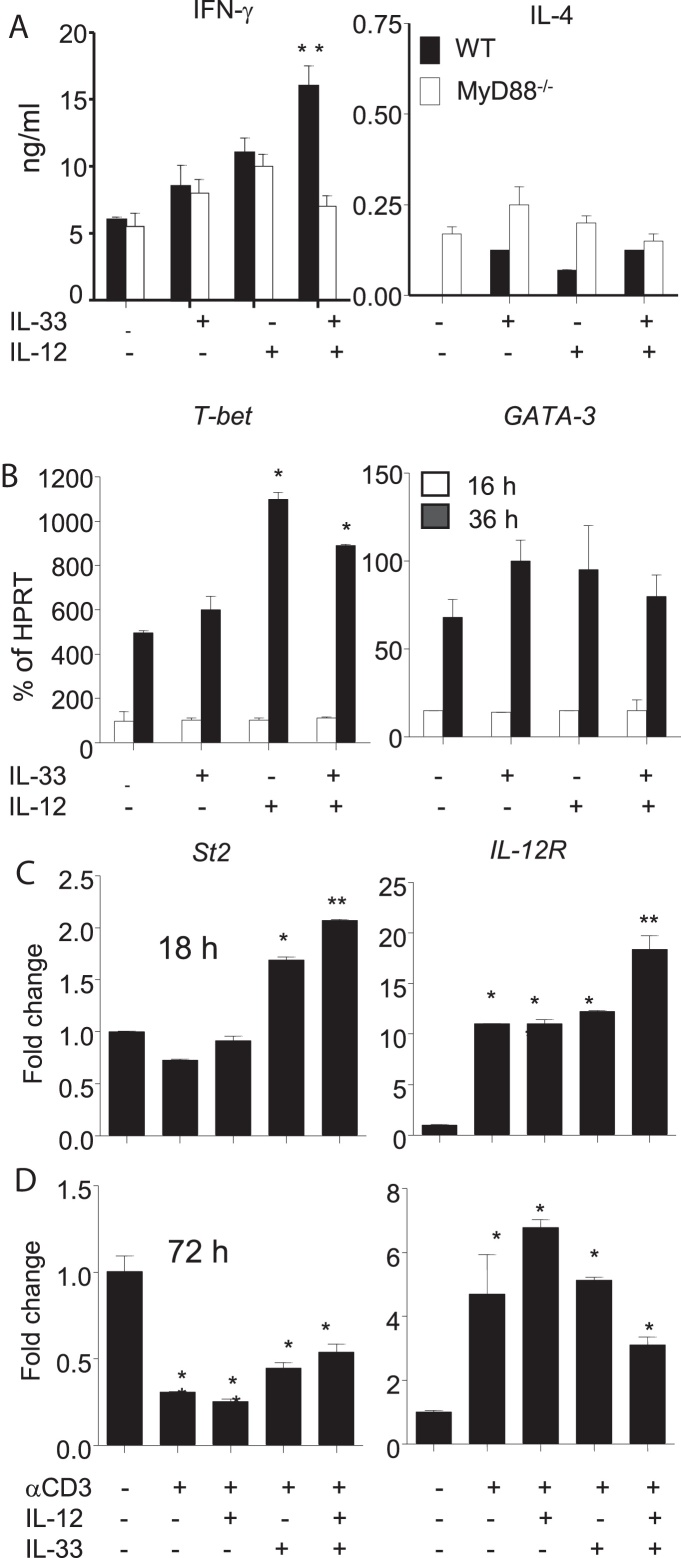
Mechanism by which IL-33 enhances Th1 cell development *in vitro*. CD4^+^ T cells from WT or MyD88^−/−^ mice were stimulated with anti-CD3 Abs in the presence or absence of IL-33, IL-12 or a combination of the cytokines. The supernatants were collected for cytokine measurement by ELISA after 72 h culture (A) and cells collected for the detection of T-bet and GATA3 (B), st2 and IL-12R (C, D) by qPCR. Data are presented as mean ± SD, *n* = 5 pooled mice/group and are representative of three independent experiments; ^∗^*p* < 0.05 compared with control, ^**^p < 0.01 compared to WT controls.

MyD88 pathway is required for IL-33 signalling (Schmitz et al., 2005). We therefore investigated the importance of MyD88 in IL-33-enhanced IFN-γ production and Th1 cell polarization in a 72 h cell culture *in vitro*. As shown in Fig. 2A, compared to WT CD4^+^ T cells, IL-33 failed to potentiate IL-12-induced IFN-γ secretion in CD4^+^ T cells from MyD88*^−/−^* mice. As expected IL-33 and IL-12 had no significant effect on IL-4 production in CD4^+^ T cells from both WT and MyD88*^−/−^* mice.

T-bet and GATA3 play a critical role in the induction and regulation of Th1 and Th2 cell development, respectively (Szabo et al., 2000, Zheng and Flavell, 1997). We next investigated the involvement of these transcription factors in IL-33 and IL-12-enhanced Th1 cell polarization and IFN-γ production in WT CD4^+^ T cells. IL-12 alone induced the transcription of T-bet but not GATA3 36 h after culture (Fig. 2B). However, IL-33 alone or together with IL-12 failed to induce or enhance both transcription factors (Fig. 2B).

We reported previously that IL-18 and IL-12 synergistically enhanced Th1 cell polarization by increasing each other’s receptor expression on CD4^+^ T cells (Xu et al., 1998a, Xu et al., 1998b). We determined whether IL-33 and IL-12 worked in a similar way in Th1 cell polarization by analysing the expression of their receptors St2 and IL-12R *in vitro*. Murine CD4^+^ T cells were activated with anti-CD3, with or without IL-12 and IL-33, individually or together, for either 18 h (Fig. 2C) or 72 h (Fig. 2D); representing the early and late Th1 cell polarization conditions, respectively. IL-33 increased the expression of St2, and IL-33 plus IL-12 synergistically enhanced both St2 and IL-12R expression after 18 h culture compared to the controls (Fig. 2C). However, after 72 h culture, the expression level of St2 after IL-33 or IL-33 plus IL-12 stimulation was significantly reduced compared to control (Fig. 2D). While the IL-12R expression levels were still higher than control, the expression levels were reduced compared with 18 h culture and further reduced in the IL-33 plus IL-12 group compared to IL-12 control group (Fig. 2D). These results therefore suggest that IL-33 and IL-12 may increase Th1 cell polarization and IFN-γ secretion in TCR-activated CD4^+^ T cells by enhancing each other’s receptor expression at the early stages of Th1 cell polarization.

### 3.3 IL-33 potentiates IL-12-mediated Th1 response *in vivo*

We next investigated whether IL-33 and IL-12 could also enhance Th1 cell development in a Th2 environment *in vivo* using the OVA/alum immunization method, a classic model for inducing Th2 response (Ma et al., 1996). C57BL/6 mice were immunized with OVA antigen in alum adjuvant, with or without IL-33 or IL-12, alone or together and challenged with OVA (protocol in methods). Spleen, draining lymph nodes (DLNs) and serum samples were collected 10 days after immunization.

**Fig. 3:**
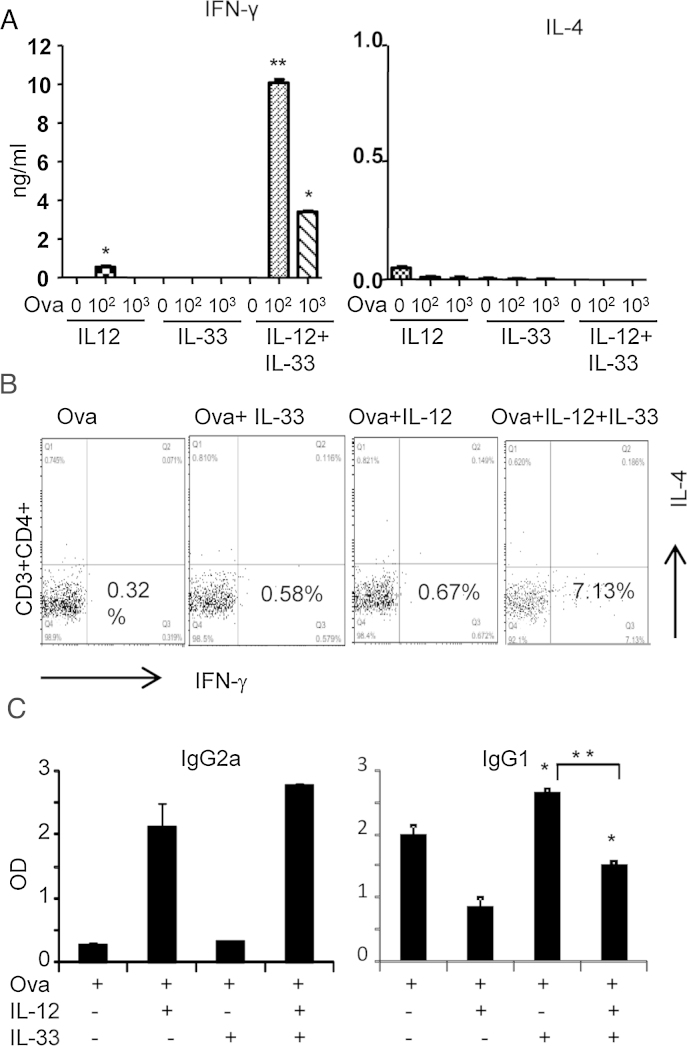
IL-33 enhances Th1 cell polarization *in vivo*. Groups of C57BL/6 mice were immunized and challenged with OVA in the presence or absence of IL-33 or IL-12, alone or together as described in Methods. The mice were sacrificed on day 10. (A) IFN-γ and IL-4 concentrations in OVA peptide-stimulated spleen cells were measured by ELISA. (B) Intracellular staining for IFN-γ and IL-4 in CD4^+^ cells from the DLN. (C) OVA-specific antibody titers in the serum samples were measured by ELISA. Data are means ± SD, *n* = 5 pooled mice/group, and are representative of three independent experiments. ^*^p < 0.05 and ^**^p < 0.01 compared to OVA controls.

We first examined the IFN-γ and IL-4 produced by spleen cells cultured *ex vivo* from groups of mice immunized with OVA antigen with or without cytokines. Spleens were harvested and single-cell suspensions were cultured with different doses of OVA peptide antigen for 48 h. Splenocytes from OVA/IL-12 group of mice produced increased concentrations of OVA-induced IFN-γ in culture compared to PBS-stimulated controls; which was further enhanced in the IL-33/IL-12/OVA group (Fig. 3A). In contrast, IL-4 was not significantly induced among the groups in this context (Fig. 3A). As expected, IL-33 alone induced high (about 700–900 pg/ml) and IL-33/IL-12 induced low (about 80–100 pg/ml) levels of OVA-specific IL-5 and IL-13 production in the culture as reported previously (data not shown and Kurowska-Stolarska et al., 2008).

We next quantified the levels of Th1 cells in the immunization experiment above. DLN cells from the groups of mice were cultured with OVA peptide antigen for 72 h and then stained for surface CD3, CD4 and intracellular IFN-γ and IL-4, respectively, *ex vivo*. OVA/IL-12 group slightly enhanced the proportion of CD3^+^CD4^+^ IFN-γ^+^IL-4^−^ Th1 cells (0.67%) in DLN cells compared to OVA (0.32%) and OVA/IL-33 (0.58%) groups (Fig. 3B). The proportion of Th1 cells in the OVA/IL-12 group was further increased in the OVA/IL-12/IL-33 group (7.13%; up to 23 fold that of the controls, Fig. 3B).

We further determined OVA-specific antibody profile in the serum samples. As shown in Fig. 3C, the OVA/IL-12 and OVA/IL-12/IL-33 groups had more specific IgG2a than the OVA and OVA/IL-33 groups (Fig. 3C). In contrast, the OVA/IL-33 group of mice produced more OVA-specific IgG1 than the OVA alone group, and this was significantly reduced in the OVA/IL-12 and the OVA/IL-12/IL-33 groups (Fig. 3C). However, no significant differences had been identified between the OVA/IL-12 and OVA/IL-12/IL-33 groups in the antibody levels in this experimental condition.

## 4 Discussion

Data reported in this study reveal a hitherto unrecognized effect and mechanism by which IL-33, a pro-Th2 cytokine, promotes Th1 cell development in human and in mice. Therefore our results may further explain its beneficial effects against tumours and infection (Bonilla et al., 2012, Gao et al., 2013, Villarreal et al., 2014) and also help explain its detrimental effects on Th1-mediated pro-inflammatory disorders (Xu et al., 2008, Verri et al., 2008).

Our results suggest that IL-33 may promote Th1 cell differentiation by the following mechanisms; (1) IL-33 signals promoting Th1 differentiation depends on IL-12 and ST2. This is evidenced by the observation that IL-33 alone cannot polarize Th1 cells, but can do so by potentiating IL-12 function in WT but not ST2^−/−^ CD4^+^ T cells. (2) It is unclear how IL-33 and IL-12 synergize in Th1 cell polarization. Our result suggests that IL-33 and IL-12 may do so by enhancing both ST2 and IL-12R expression in early activated CD4^+^ T cells (Fig. 2C). Furthermore, we found that the enhanced ST2 expression induced by IL-33 and IL-12 was down-regulated 72 h after Th1 polarization *in vitro*. This is agreed with the previous report that ST2 expression during late Th1 cell development was decreased *in vitro* and *in vivo* (Baumann et al., 2015) and that established Th2 but not Th1 cells express ST2 (Xu et al., 1998a, Xu et al., 1998b, Löhning et al., 1998). These findings suggests that ST2 expression is induced during early Th1 cell polarization in naïve CD4^+^ T cells by IL-33 and IL-12 signalling which is gradually inhibited when Th1 cells fully mature (Fig. 3 and Xu et al., 1998a, Xu et al., 1998b). Thus, while it may promote Th1 cell development, it is unlikely that IL-33 has profound effect on mature Th1 cells. (3) The signalling pathway by which IL-33 enhances Th1 polarization is still unknown. We found that MyD88 is required for the IL-33 and IL-12-induced Th1 cell development and IFN-γ production. It is also reported that T-bet but not GATA3 is involved in the IL-33-promoted Th1 cell polarization (Baumann et al., 2015). We also found that IL-12 alone or together with IL-33 enhanced T-bet but not GATA3 expression in TCR-activated CD4^+^ T cells. More studies are needed to understand the precise signalling pathway that is involved in IL-33-potentiated Th1 cell development.

IL-12 and IL-4 reciprocally regulate Th1 or Th2 cell differentiation and function (Sher and Coffman, 1992, Constant and Bottomly, 1997, O’Garra et al., 1997). It is likely that IL-33 inducing Th1 or Th2 response depends on the cytokine milieu, in particular the balance of IL-12 and IL-4 *in vivo*. As such, in the IL-12-dominant cytokine milieu, for instance, in the pro-inflammatory conditions or bacterial/virus infection, secreted IL-33 may potentiate IL-12-mediated Th1 cell differentiation (Constant and Bottomly, 1997; Boumann et al., 2015). In the IL-4-dominant cytokine milieu, for example, in allergy or parasite infection, IL-33 may preferably promote Th2 cell development and function (Schmitz et al., 2005, Kurowska-Stolarska et al., 2008, Komai-Koma et al., 2012). Therefore, IL-33 is a pleotropic cytokine in Th1 and Th2 cell development and contributes to both Th1 and Th2-mediated immunity and disorders depending on cytokine milieu.

While enhanced IL-12-mediated Th1 polarization *in vivo*, we found that IL-33 could not further potentiate IL-12-induced antibody profile in this experimental condition. This may be because IL-33 alone can also polarize a Th2-like cell set which only produces IL-5 and IL-13, but not IL-4 as reported previously, and this cell set is capable to induce Th2-like response *in vivo* (Kurowska-Stolarska et al., 2008).

It is reported that IL-33 and IL-12 also synergistically enhances Tc1 cell development in CD8^+^ T cells (Yang et al., 2011). However, the effect and mechanism by which IL-33 enhances Th1 cell function in our study differs from that in Tc1 cells in at least two aspects; (1) IL-33 promotes Th1 differentiation from naïve T cells in our experiment but not Tc1 cell differentiation as in the previous report (Yang et al., 2011). (2) IL-33 can enhance established effector Tc1 but not mature Th1 cell function (Yang et al., 2011). The likely explanation for these differences is because ST2 expression is induced only in early TCR activated naïve CD4^+^ T cells which is then gradually inhibited when Th1 cells fully mature (Fig. 3 and Xu et al., 1998a, Xu et al., 1998b). In contrast, ST2 is only expressed on effector CD8^+^ T cells or polarized Tc1 cells but not on naïve and early activated CD8^+^ T cells (Yang et al., 2011). Nevertheless, these findings highlight the potential importance for IL-33 to enhance an antigen-specific type 1 response by promoting both Th1 and Tc1 cell function in adaptive immunity and in inflammatory disorders.

The clinical relevance of the IL-33-enhanced Th1 cell development in immunity and disease is unknown. Since IL-33 is induced by many pathogens and inflammatory agents this novel Th1-development pathway may play an important role in a wide-range of infectious and inflammatory disorders. Furthermore, IL-33 may also serve as a novel adjuvant in vaccination against cancer and infectious diseases.

## Funding

This study received financial support from Arthritis Research UK (MP/18912 to D.X.).

## Conflict of interest.

The authors have no financial conflicts of interest.
